# An Exploratory Study on the Acoustic Musical Properties to Decrease Self-Perceived Anxiety

**DOI:** 10.3390/ijerph19020994

**Published:** 2022-01-16

**Authors:** Emilia Parada-Cabaleiro, Anton Batliner, Markus Schedl

**Affiliations:** 1 Multimedia Mining and Search Group, Institute of Computational Perception, Johannes Kepler University Linz (JKU), 4040 Linz, Austria; markus.schedl@jku.at; 2 Human-Centered AI Group, AI Laboratory, Linz Institute of Technology (LIT), 4040 Linz, Austria; 3 Embedded Intelligence for Health Care and Wellbeing, University of Augsburg, 86159 Augsburg, Germany; anton.batliner@informatik.uni-augsburg.de

**Keywords:** music psychology, audio features, self-report, signal processing, induced distress, every-day anxiety

## Abstract

Musical listening is broadly used as an inexpensive and safe method to reduce self-perceived anxiety. This strategy is based on the *emotivist* assumption claiming that emotions are not only recognised in music but induced by it. Yet, the acoustic properties of musical work capable of reducing anxiety are still under-researched. To fill this gap, we explore whether the acoustic parameters relevant in music emotion recognition are also suitable to identify music with relaxing properties. As an anxiety indicator, the positive statements from the six-item *Spielberger State-Trait Anxiety Inventory*, a self-reported score from 3 to 12, are taken. A user-study with 50 participants assessing the relaxing potential of four musical pieces was conducted; subsequently, the acoustic parameters were evaluated. Our study shows that when using classical Western music to reduce self-perceived anxiety, tonal music should be considered. In addition, it also indicates that harmonicity is a suitable indicator of relaxing music, while the role of scoring and dynamics in reducing non-pathological listener distress should be further investigated.

## 1. Introduction

According to the American Psychological Association, “anxiety is an emotion characterized by feelings of tension, worried thoughts, physical changes, and increased blood pressure” [1]. In modern Western society, anxiety is often considered a normal condition suffered everyday. Triggered by the fear of a variety of events, such as threats to public safety, anxiety affects both individuals and social entities, and its consequences vary considerably between the former and the latter [2].

Although pharmacological treatment and cognitive-behavioural therapy are well established procedures to deal with anxiety disorders [3], the use of music as a non-invasive intervention to regulate daily stress [4] is increasing; its suitability is generally acknowledged in both medical and non-medical settings [5]. To explain the mechanisms behind listening to music as a medium to decrease anxiety, a variety of theories have been presented, including music’s capability to trigger pleasant memories [6], counterbalance negative feelings [7,8,9], and mask unpleasant and stressful noise [10].

Concerning the musical properties involved in this phenomenon, previous work identified slow tempo, low pitch, string timbre, rhythm regularity, or the absence of lyrics as generally suitable parameters to reduce anxiety by listening to music [11]. Due to its generally known positive properties, listening to music on a daily basis is indeed a common strategy followed by non-clinically diagnosed individuals to cope with quotidian distress [12]—a strategy that has shown to be particularly suitable when deliberately chosen for relaxing purposes [13].

Although listening to music on a daily basis appears to reduce self-perceived anxiety, the variety of methodologies considered in the literature, employing, for instance, different types of music and participants, has led, in some cases, to contradictory outcomes, which impedes a clear understanding and generalisation of the existing results [14]. From the great diversity of evaluated musical genres, classical Western music, unlike others, such as folk [15] and electronic [16,17], has been the most commonly evaluated in prior work [11,18,19,20] and is generally accepted as efficient in reducing anxiety [5] within the WEIRD (Western, educated, industrialized, rich, and democratic) population [21]. Note that by ‘classical’, we refer to music that is not light/popular (cf. 4th definition in [22]); however, this should not be confused with the music from the classical period, i.e., post-Baroque and pre-Romantic (cf. first definition in [22]).

Concerning the types of anxiety, amongst those considered in the literature when evaluating the effect of music, the most salient ones are medical-related anxiety and induced anxiety. In the former, experiments are carried out in hospitals and typically involve perioperative processes [23,24] and childbirth [25,26]. Differently, induced anxiety is commonly considered by researchers outside of healthcare infrastructures, who evoke low aroused states of distress in ‘typical’ individuals through challenging tasks, such as mental arithmetic exercises [27,28], or by creating psycho-social stressful situations, such as public speaking [29,30].

Finally, it is also important to mention that music’s ability to evoke listeners’ emotional reactions involves underlying mechanisms, such as the *evaluative conditioning* [31]: A piece of music might reduce anxiety for a specific listener because it was previously paired with relaxing stimuli. Related to this mechanism are listeners’ familiarity and preferences that, together with stereotypes linked to specific musical genres [32], have shown to influence listeners’ responses to music [33,34] and therefore music’s relaxing effects.

The use of computational methods in emotion-, mood-, and sentiment-related research has marked a milestone in the understanding of human psychological states; *Affective Computing* [35,36] is currently a major research field in AI at the intersection between computer science, psychology, and cognitive science. Similarly, the application of computational methods and signal processing techniques is becoming increasingly common in the understanding of emotions in music, as shown by the unprecedented growth in the field of Music Emotion Recognition (MER) [37].

Nevertheless, previous works aiming to identify the acoustic features involved in transmitting affects through music often refer to the 2-dimensional model of emotions centred on valence and arousal [38,39,40,41]; this conceptualisation has been strongly criticised for being insufficient when discriminating between some emotional categories [42].

Indeed, according to Russell’s circumplex model [43], emotional adjectives related to the concept of anxiety, such as alarmed, afraid, or tense, are very close to anger in the dimensional space. In addition, even in the studies that consider the categorical model [44], anxiety is not an emotional state taken into account when identifying the acoustic features related to emotion.

In this regard, it is worth mentioning that most of the previous works investigating the acoustic emotional properties of a musical piece [39,40,41,44] refer often to the ‘perceived emotions’, i.e., the emotions that, from a listener’s point of view, are expressed by a musical piece, according to the *cognitivist position* [45]. This differs from the ‘induced’ or ‘felt emotions’, i.e., the emotions induced through a music piece and experienced by the listener, according to the *emotivist position* [46].

Apart from rare attempts of modelling musical emotions through a meta-level framework combining both positions [47], from a signal processing perspective, the relationship between music and emotion has often been assessed according to the *cognitivist position* [39,40,41,44], while the *emotivist* one is still under-researched [38]. When investigating the musical acoustic properties involved in reducing users’ self-perceived anxiety, considering the *emotivist position* and the categorical model of emotions becomes particularly important.

The former, since we are interested in assessing how the user feels [46]; the latter, because the dimensional model cannot clearly discriminate between anxiety and other negative emotional states [42]. Thus, even though the acoustic properties involved in evoking listeners’ felt emotions in terms of arousal and valence have been investigated [38], to which extent these are also applicable when modelling anxiety remains unclear.

Given this background, the aims of this exploratory study are threefold:

(i) To investigate the relaxing properties of four contrasting musical samples from different Western historical traditions. Two of these traditions have already been investigated in previous works [11,20], i.e., *Baroque* and *Impressionism*; two are still under-researched, i.e., *Gregorian chant* and *Expressionism* (the latter two chosen for their contrasting characteristics with respect to the former two). In order to assess low intensity states of anxiety that might be more common in every-day situations, anxiety induced through Mood Induction Procedures (MIP) was preferred to the medical one—note that, through MIP, only low aroused emotions should be elicited [48].
(ii) To assess whether music with the capability to reduce users’ self-perceived (induced) anxiety acoustically differs with respect to that without such a capability. For this, well-established audio feature sets tailored to emotional modelling in the context of speech and music processing are taken into account [39,49,50]. Note that feature sets from both domains are considered since speech and music are communication channels that share the same acoustic code for expressing emotions [41,51].
(iii) To connect the massive research on the treatment of anxiety from music psychology and music therapy with the continuously increasing studies on emotion from Music Information Retrieval (MIR), in particular MER. This connection will be highly beneficial in the identification of the musical and acoustic properties suitable to reduce listeners’ anxiety.

The rest of this article is laid out as follows: Section 2 summarises the materials and methods through the description of the musical stimuli (Section 2.1), the anxiety induction and measurement (Section 2.2), the user study (Section 2.3), and the acoustic features (Section 2.4). Section 3 presents the results for both, the user study (Section 3.1) and the acoustic evaluation (Section 3.2). Section 4, gives our discussion. Finally, Section 5 concludes the manuscript.

## 2. Materials and Methods

### 2.1. Musical Stimuli

The effect of classical Western music in reducing anxiety has often been investigated for non-clinical data [52], and its suitability within the WEIRD population is generally acknowledged in both clinical and non-clinical settings [5,11]. In particular, when evaluating induced anxiety, classical music produced statistically significant relaxing effects in the listeners disregarding their personal preferences [53]. Studies have been presented displaying positive effects even in non-Western individuals [54].

Due to the great diversity across classical compositions from different historical periods, four samples written in four contrasting ‘styles’ concerning different composition principles, such as the use of a tonal centre, rhythm regularity, scoring, and orchestration techniques, were selected. As in previous work [19,55], samples of around five minutes were considered.

Due to contradictory study outcomes concerning the role of users’ musical preference when using music to reduce anxiety [7,11,19], and to avoid further complexity, listeners’ musical preference was not explicitly considered. Note that, given the dominance of tonal music in Western culture, we assume that the more defined the tonal centre of the musical piece is, the more familiar this would be to the listener.

**Canon in D major (Pachelbel):** This *Baroque* musical sample, characterised by its regular rhythm, gentle melodic contours, tonal harmony, and string timbre, has often been used in previous research on anxiety reduction [20,30,55,56,57]. A historically informed performance, observing the principles from the time when the composition was written, such as temperament and ornamentation, by the ensemble *Voices of Music* [58] was considered. Due to its clearly defined tonal centre, we assumed that this is the sample most familiar to the listeners, who even without a formal education in Western music would have been exposed to Baroque music, typical in advertisements and other Western media.

**Credo (Gregorian Chant):** The Credo is a prayer of the Mass’ Ordinary typically set to music [59]. An *a capella* interpretation by Marek Klein, recorded within the *Graduale Project*, which follows the *Graduale Triplex*, was considered. The chant, identified as Credo 3, is accessible in the recordings of the fourth year (second part, 2016) in Amazon Music. Gregorian chant has been developed as an expression of spirituality [60], and  still today, this chant, characterised by its simplicity and soft contours, continues to be used in support of meditation—a practice whose effects in decreasing anxiety are still an object of investigation [61,62].

Although the use of Gregorian chant might not strictly follow the general rule of refraining from using music with lyrics to reduce anxiety [11], we consider that the lyrics would not particularly affect the listeners mainly for two reasons.

First, their meaning (in Latin) would be hardly understood by the listeners. Considering non-understandable lyrics is indeed a strategy to avoid their influence when using music with relaxing purposes [53].

Second, as the concepts expressed by the lyrics have a contextualised meaning only within the liturgy, we assume that passively listening to the lyrics of the Gregorian chant out of context would not influence the participants. This sample, being modal, presents a lower degree of familiarity to the listeners with respect to Pachelbel’s canon.

**Prélude à l’après-midi d’un faune (Debussy):** The *Prelude to the afternoon of a faun* shows the typical characteristics of the *Impressionist* movement in music [22,59]: static melodic contours; delicate textures that blur the orchestra in colourful instrumental combinations; a vague conceptualisation of the form, split superficially in large sections, which is constantly evolving; and an ambiguous use of the tonality, based on chords of the 9th, 11th, and 13th, parallel movements, and exotic scales.

This piece was selected as another contrasting sample that has also been considered to reduce anxiety in previous works [11,63]. A performance by the *Orchestre symphonique de Montréal*, conducted by Charles Dutoit, was considered. The recording by Decca (2004) is accessible at Amazon Music. The recording was cut with a fade-out after the first half of the bar 52 (at minute 4:53), which, due to the perfect cadence onto A-flat major, i.e., the first strong resolute cadence in the piece, constitutes a natural inflexion point of the musical sample. As with the Gregorian chant, this sample also presents a lower degree of familiarity to the listeners with respect to Pachelbel’s canon.

**Vergangenes—Fünf Orchesterstücke (Schönberg):** The second piece, *Vergangenes* (The past), from the *Five pieces for orchestra* is characterised by extreme contrasts of dynamics, texture, and instrumentation, as well as by vague tonal references distorted by the avoidance of traditional harmonic conventions, as shown by an emphasis in the use of dissonances and tritones [59]. This piece, which can be considered a representation of the *Expressionism* movement in music [22], was selected as a strong contrast to the others since it is the only one written in a pure chromaticism language, i.e., quasi atonal. A performance by the *Cleveland Orchestra*, conducted by Christoph von Dohnányi, was considered. The recording by Decca (1997) is accessible in Amazon Music. Due to its intentional avoidance of the tonal language, we assumed that this is the sample least familiar to the listeners, who—without any formal education in Western music—would have been little exposed to this kind of musical language.

### 2.2. Anxiety Induction and Measurement

To increase the ecological validity of the study [52], following similar studies [7,29,53], the experimental procedure started with a demanding task aimed to induce a similar level of anxiety in all the participants. For this, the *Stroop Color and Word Test* (SCWT), a standard procedure in the investigation of methods for anxiety reduction, such as anxiolytic drugs [64] or music listening [28], was considered. The SCWT is a neuropsychological test that evaluates the users’ capacity to inhibit the *Stroop Effect* [65], a phenomenon that occurs when a specific stimulus’ feature impedes the simultaneous processing of another attribute from the same stimulus [66].

**Figure 1:**
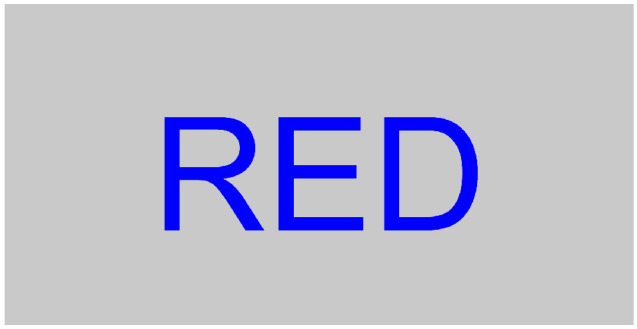
Colour–word pair from the *Stroop Color and Word Test*. Users should denote the colour of the ‘coloured’ word i.e., ‘blue’, instead of reading the word ‘red’.

It is based on one of the cognitive conflicting tasks presented by Stroop [67], where users have to name a colour of a ‘coloured’ word that denotes a different colour (colour–word pair), i.e., the naming task. Note that this is different to the reading task, where the text should be read. Five colours (blue, yellow, red, green, and pink) were considered, yielding 10 colour–word pairs (cf. one pair in Figure 1).

Following Teixeira-Silva et al. [64], the induction procedure was performed through a 2-minute-video integrated in the smartphone-based interface. The video was created by randomly presenting a different colour–word pair per second. To increase the pressure, the task was presented as an interactive voice-driven application, i.e., the participants were instructed to pronounce the colour. To make the procedure realistic, a ‘fake’ permission to access users’ microphone was required. They were also instructed to answer as fast as possible in order to achieve a good score. The task was intentionally performed in English to increase its difficulty (note that only five participants were native English speakers). However, to guarantee comparability across users, a minimum proficiency level of B1 was required to carry out the test.

To measure the participants’ anxiety, we consider users’ self-reports, a standard indicator that has proven to be suitable [52]. As measurement procedure, we chose an adaptation of the six-item short-form of the *Spielberger State-Trait Anxiety Inventory* (STAI: Y-6 item [68]), a self-perception-based instrument extensively used to assess anti-anxiety interventions [69], particularly when investigating the effect of musical treatments [10,11,20,29,30,52,63,70,71]. The STAI: Y-6 scale encompasses six statements rated by the user according to a four-point-Likert scale, from  1 (not at all) to 4 (very much): three out of the six statements are positive and three negative.

As only the positive statements yielded meaningful results in [55], only these were assessed: ‘I feel calm’, ‘I am relaxed’, and ‘I feel content’; thus, the overall score for each user ranged from 3 to 12. To quantify stress relief, we computed score differences between pre- and post-treatment, i.e., the differences between the STAI scores obtained before and after listening to the music (see Section 3.1).

### 2.3. User Study

The ‘relaxing’ effect of each of the four musical samples was evaluated with respect to the control conditions, in which no music was played. The experiment was carried out on a smartphone-based interface hosted on the platform *Typeform* [72] and the participants were instructed to wear headphones. In order to generalise to an extent the outcomes of the user study, an heterogeneous group of participants was recruited from different ethnicities, as considered in a similar study [53].

Note that, since we are not interested in a homogeneous group, collecting information about the users’ ethnicity was only considered for descriptive reasons, but it was not a mandatory question. The participants were recruited by the authors who advertised the experiment through their social networks. This was considered the most efficient strategy to guarantee a heterogeneous sample. The distribution of participants across country of origin is as follows: Spain (14), Serbia (7), Italy (4), Germany (4), China (7), UK (2), India (3), Israel (2), The Netherlands (1), and undeclared (6).

All the participants had been exposed to Western musical culture; the non-caucasian participants were all international university students established in European countries for more than three years. Since the vast majority of participants did not have a formal background in classical Western music (only six of them had received institutionalised training), we generally assume that Pachelbel’s canon was the most familiar sample to all and that the one by Schönberg was the most unfamiliar.

To assign the participants to each group, an initial set of 75 candidates was created by the authors from their social network. The candidates were automatically distributed randomly across the five groups; afterwards, they were manually ranked by attempting to prioritise a balanced distribution across groups in terms of gender, age, ethnicity, and musical background. Subsequently, the ranked candidates were contacted until the criterion of 50 participants distributed across five groups was met.

After collecting the users’ demographics and consent concerning the use of their anonymised responses for research purposes, the anxiety induction procedure was carried out; subsequently, users’ anxiety was measured through the STAI: Y-6 scale. This was followed by the experimental condition: Participants in the treatment groups listened to a musical sample; those in the control group stayed in silence for 5 min. Again, users’ anxiety was measured. To evaluate the effect of each musical treatment with respect to the control conditions (cf. Section 3.1), the difference between the first anxiety measurement (pre-experiment) and the second (post-experiment) was considered.

To mitigate the *demand effect*, i.e., the influence of the users’ understanding of the experimental purpose on the induction procedure, users were told that their performance in the *Stroop Color and Word Test* would be evaluated with respect to demographic information, such as nationality, gender, and age. Hiding the real purpose of an experiment to avoid a bias in the users’ behaviour is indeed a common strategy when inducing emotional states, such as anxiety [48,73].

For the evaluation, univariate analysis of variance (ANOVA) was applied, since, for the assumptions homogenetity and normality, the Null-Hypothesis (H0) was confirmed: for the homogeneity test, Levene yields F =0.6515, p=0.628; for the normality assessment, Kolmogorov–Smirnov yields D =0.16, p=0.544. The Tukey post-hoc test was chosen to carry out the multiple comparisons considering as a reference the control group. Since Null-Hypothesis-Testing with *p*-values as decisive criterion has been repeatedly criticised [74], we report *p*-values as a standard descriptive measure; the outcomes of the statistical analysis will be interpreted in terms of effect size [75]: $\eta^{2}$ for the ANOVA; and Cohen’s *d* for the pairwise comparisons.

### 2.4. Acoustic Features

To the best of our knowledge, in the realm of signal processing, no studies computationally investigating the acoustic features with the capability to reduce listeners’ anxiety have been conducted thus far. As a first attempt to fill this gap, the capability of four acoustic feature sets in discriminating between musical pieces with different relaxing properties were investigated: EmoMusic, ComParE, eGeMAPS, and NoAnx (cf. Table 1). On one side, the acoustic properties already identified in previous works as suitable to express emotions [39,76] and to reduce listeners’ pain [77] were considered: we will refer to these as the EmoMusic feature set.

On the other side, in order to identify other acoustic features potentially suitable in reducing listener’s anxiety, two additional feature sets, tailored to model emotional content from audio sources, were considered: ComParE (Computational Paralinguistics ChallengE) [49] and eGeMAPS (extended Geneva Minimalistic Acoustic Parameter Set) [50].

To evaluate whether the differences between relaxing properties are mirrored by the differences between acoustic representation, the best-performing features of the three feature sets in differentiating between the evaluated musical samples were identified employing Principal Component Analysis (PCA). For this, the features with a Pearson correlation |r|>0.5 with respect to at least one of the top two PCs (first and second), i.e., a moderate/high correlation [78], were considered. The union of these features, referred to as NoAnx, was evaluated as an additional feature set (cf. Section 3.2).

For each musical sample, acoustic Low Level Descriptors (LLDs) were extracted with the OpenSMILE toolkit [79]. Concerning the frame size, the default optimal configuration was kept (60 and 25 ms, depending on the specific LLD); differently, to  avoid redundant information due to the overlap between frames (which would have biased the statistical evaluation), and, in order to preserve equal lengths across feature vectors (necessary to carry out the PCA), a fixed hop size of 60 ms without overlap was considered for all the LLDs. Although this implies that acoustic information is not continuously captured for some LLDs, we considered it as a reasonable compromise to prevent redundancies and keep the data format consistent.

The acoustic features from EmoMusic, described in [39,76], encompass 8 LLDs: roll off, sharpness, spectral centroid, harmonicity, energy, loudness, F0, and spectral flux; these 8 LLDs were taken from the ComParE [49] feature set. Those from ComParE, described in [41,80], encompass 65 LLDs including Mel-Frequency Cepstral Coefficients (MFCCs), spectral features, prosodic features, and sound quality features. The ones from eGeMAPS, described in [50], encompass 25 LLDs, including frequency-related features, energy/amplitude related parameters, and spectral features. Note that the following seven descriptors are common in ComParE and eGeMAPS: *spectral flux*, *mfcc* (1 to 4), *jitter*, and *shimmer*.

**Figure 2:**
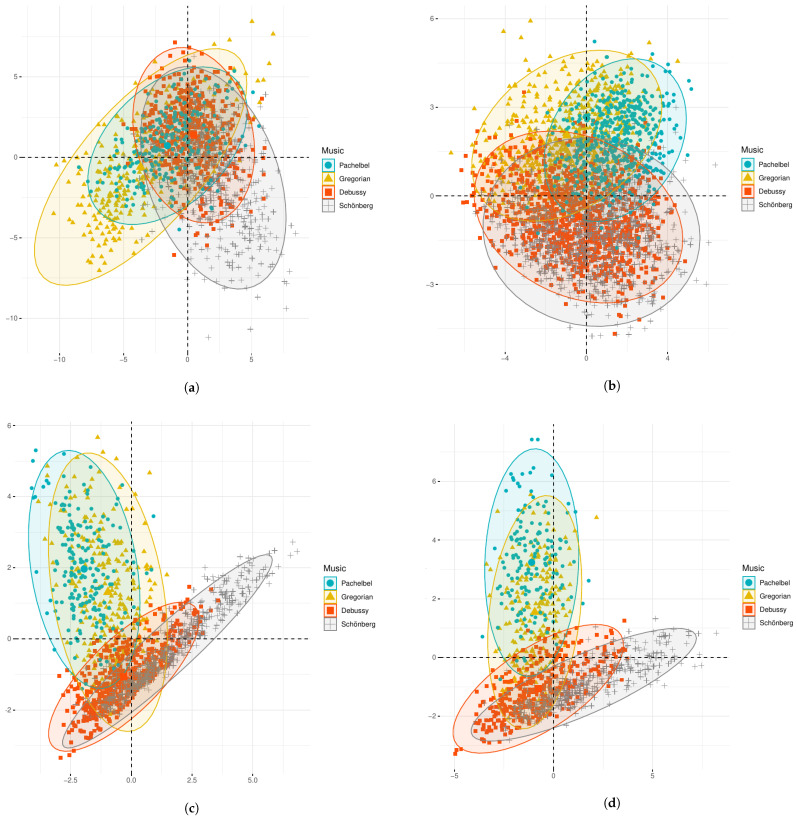
Principal Component (PC) representation for the LLDs in ComParE, eGeMAPS, EmoMusic, and  NoAnx. Constellations for each sample: Pachelbel’s canon, Gregorian chant, Debussy’s sample, and Schönberg’s sample, are shown. (**a**) First and second PCs for ComParE. (**b**) First and second PCs for eGeMAPS. (**c**) First and second PCs for EmoMusic. (**d**) First and second PCs for NoAnx.

In order to gain further insight on how the considered feature sets are capable of discriminating between the evaluated musical samples, in Figure 2, the representation of the four samples is shown. Note that the number of data points varies across samples and feature sets as it depends on one side on the samples’ length and, on the other side, on the amount of multicollinear outliers that have been removed. The redundancy of some features yields a high overlap between the feature maps across the four pieces.

This is especially evident for ComParE (cf. Figure 2a), whose acoustic representation is condensed around the central area, showing a large overlap of Pachelbel’s canon with respect to the other pieces. EmoMusic enables a better discrimination between the musical samples than ComParE and eGeMAPS, presenting mainly two confusion patterns: Pachelbel vs. Gregorian, Debussy vs. Schönberg (cf. Figure 2c).

This confusion can be explained, to some extent, in terms of scoring: the Gregorian chant and Pachelbel’s canon both present a reduced timbre variety due to the use of an *a capella* single voice (the first) and a small baroque ensemble (the second); the samples by Debussy and Schönberg present both a rich sonority, due to the use of orchestral timbre in both cases.

Since this confusion pattern is also observed for eGeMAPS (cf. Figure 2b), the LLDs from eGeMAPS not contained in EmoMusic, i.e., three LLDs, were added to the latter, by this creating NoAnx. These three additional LLDs are: the alpha ratio (ratio of the summed energy from 50–1000 Hz and from 1–5 kHz); the Hammaberg index (ratio of the strongest energy peak from 0–2 kHz with respect to the strongest peak from 2–5 kHz), and  the MFCC2 (second mel-frequency cepstral coefficient).

All in all, the  11 LLDs of NoAnx (8 from EmoMusic + 3 from eGeMAPS), refer to three musical-perceptual properties: *Timbre*, related to the tonal quality or colour of the sound [81], acoustically represented by roll off, sharpness, spectral centroid, harmonicity, and MFCC [50]; *Dynamics*, related to the perceived intensity and sound pressure level, represented by energy and loudness [81]; and *Pitch*, related to the changes in the power spectrum of a signal over time and to the melodic contour, represented by F0, spectral flux [76], alpha ratio, and Hammarberg index [50]. The feature maps from NoAnx show a higher dispersion from the central area of the bi-dimensional space, a tendency more prominently displayed for Pachelbel’s canon and Schönberg’s sample, with  no overlap between them (cf. Figure 2d).

## 3. Results

### 3.1. User Study

A total of 50 subjects (29 female, 21 male), with ages from 18 to 67 years (μ=31.6, σ=9.4) participated in the study: Asian (10), Caucasian (34), and undeclared (6). To evaluate the effect of the musical samples in reducing listeners’ self-perceived anxiety, the differences in user responses between pre- and post-treatment, i.e., the difference between before and after listening to each evaluated musical sample, were compared with those from the control group (users who did not listen to any music).

The mean and standard deviation (μ±σ) in pre- and post-treatment for the control group and each treatment condition are reported in the following: Control group (pre =7.4±1.7, post =7.6±2.2); Pachelbel’s canon (pre =6.3±1.7, post =9.5±1.3); Gregorian chant (pre =7.6±2.7, post =9.1±2.3); Debussy’s sample (pre =7.1±1.9, post =8.3±2.5); Schönberg’s sample (pre =6.3±2.4, post =6.9±2.2).

The one-way ANOVA showed statistically significant differences between group means, as indicated by the medium-large effect size: F(4,45) =3.314, p=0.018, $\eta^{2}=0.23$. The pairwise comparisons indicate that the only musical sample that can be associated with a positive effect in reducing self-perceived anxiety is the Pachelbel’s canon, as shown by the medium effect size (cf. d=0.55 in Table 2). Indeed, this is the only treatment condition that yielded a positive lower confidence interval (CI): lwr =0.45 (cf. Pachelbel in Table 2), indicating that, unlike the other musical samples, Pachelbel’s canon contains properties that can be related to a decline in self-perceived anxiety.

In contrast, the sample by Schönberg yielded the smallest mean difference and the greatest (negative) lower CI with respect to the control group: diff =0.4, lwr =−2.15 (cf. Schönberg in Table 2). Values for the Gregorian sample and the one by Debussy were in between. This might relate to the level of familiarity of the listeners with the musical language: Pachelbel’s canon (in tonal language) is familiar to many listeners, as shown in prior work [55]; the sample by Schönberg (almost atonal language) is unfamiliar to most; the Gregorian sample and the one by Debussy (based on modal scales) fall between the other two in terms of familiarity.

### 3.2. Acoustic Features

Since all the features contained in NoAnx (cf. Section 2.4 and Figure 2d) presented a moderate/high correlation with respect to the two first PCs, i.e., Pearson’s |r|>0.5 [78], these will be further evaluated. In addition, considering that the only musical sample that can be associated with a decline in listeners’ anxiety was the Pachelbel’s canon, in order to assess whether there is a difference between the acoustic representation of this sample and the one for the other three, a one-way-ANOVA was carried out for each feature taking Pachelbel’s canon as reference.

Since the homogeneity assumption was violated, Welch-ANOVA was performed [82] with Games–Howell post-hoc for non-parametric pairwise comparisons. Again, the results were evaluated in terms of effect size [75]: epsilon squared ($\epsilon^{2}$) for the Welch-ANOVA, Hedge’s *g* for the multiple comparisons. Although  we performed an individual ANOVA for each feature, multicollinear outliers across features were identified (and subsequently removed) by computing the Mahalanobis distance [83]. Performing individual ANOVAs was preferred to a multivariate analysis of variance (MANOVA), since the correlations between features was either |r|>0.9 or |r|<0.2, which makes the MANOVA unsuitable [84].

In Table 3, the results for the Welch-ANOVA and the multiple comparisons between Pachelbel’s canon and the other three musical samples are presented. Substantial differences between conditions are shown, as indicated by the medium ($0.10\leq \epsilon^{2}\leq 0.28$) and large ($\epsilon^{2}\geq 0.57$) effect sizes, for all the features; cf. $\epsilon^{2}$ in Table 3. The greater differences (shown for harmonicity, dynamics, and spectral flux) are most prominently displayed with respect to the samples by Debussy and Schönberg, less with respect to the Gregorian chant. These differences can be also interpreted in musical terms. Pachelbel’s canon, characterised by the use of tonal functions, shows a higher level of harmonicity than the others samples, specially the one by Schönberg (cf. highest diff =−0.43 for harmonicity, Schönberg, in Table 3).

Similarly, Pachelbel’s canon presents a ‘stability’ and ‘continuity’ conferred by the use of *basso continuo*, which likely played a role in the higher levels of dynamics in comparison to the sample by Debussy but particularly to the one by Schönberg, characterised by the exploration of orchestral timbre effects rather than the dynamics (cf. highest differences: diff =−0.05 and diff =−0.97 for dynamics, Schönberg, in Table 3).

Concerning the spectral flux, although this feature is hardly interpretable, the higher levels for Pachelbel’s canon might be due to aspects related to instrumentation and articulation. On one side, the timbre of a string orchestra is in contrast with the use of also winds (samples by Debussy and Schönberg) and singing voice (Gregorian chant); on the other side, the regular and marked rhythms of the former are in contrast with the blurry transitions across sonorities particularly shown for the orchestral samples, i.e., those by Debussy and Schönberg.

We interpret that these two aspects have lead to higher spectral changes between successive audio frames for Pachelbel’s canon than for the other samples (cf. highest diff =−0.31 for spectral flux, Schönberg, in Table 3).

For the other features, the differences with respect to the Gregorian chant and Debussy’s sample, both musically closer to Pachelbel’s canon than Schönberg’s sample, are generally lower (cf. diff for Gregorian and Debussy in Table 3). This mirrors the results for the listeners’ responses in terms of self-perceived anxiety: Pachelbel’s canon can be associated with an observed reduction in listeners’ self-perceived anxiety; Schönberg’s sample seems not to show any relationship to listeners’ self-perceived anxiety (thus, showing marked differences with respect to Pachelbel’s canon for almost all the acoustic features); Debussy’s sample and the Gregorian chant are in between (cf. Section 3.1 and Table 2).

**Figure 3:**
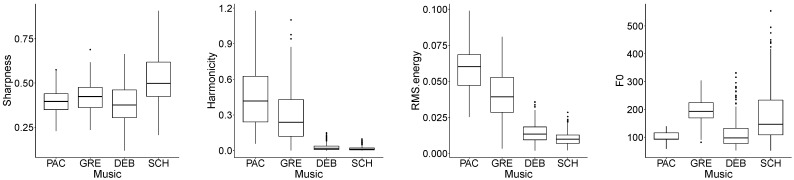
Representation of the musical samples (*x*-axis): Pachelbel (PAC), Gregorian (GRE), Debussy (DEB), and Schönberg (SCH); for the acoustic features (*y*-axis): sharpness, harmonicity, RMS energy, and F0; from left to right, respectively. First quartile, second quartile, median, third quartile, fourth quartile, and outliers (from bottom to top) are indicated by the box plots.

In order to have a closer look at some of the most interesting results, in  Figure 3, box plots for four selected features are displayed. Concerning the timbre-related features, all of them show a progressive increase from Pachelbel’s canon to Schönberg’s sample (Gregorian chant and Debussy’s sample in between) except for harmonicity, which shows the opposite trend; cf. sharpness and harmonicity, respectively, in Figure 3. In musical terms, harmonicity and sharpness are often inversely related; for instance, tonal music, such as Pachelbel’s canon, due to its typical consonant chord progressions, soft melodic contours, and predictable interval relationships, shows a very high level of harmonicity and low level of sharpness; music intentionally avoiding a tonal centre, such as Schönberg’s sample, shows the opposite trend.

Concerning the dynamics-related features, as already mentioned, the orchestral pieces, i.e., the ones by Debussy and Schönberg, are those showing a lower energy level (cf. DEB and SCH for RMS.energy in Figure 3). This can be explained, as indicated, with the type of orchestration that employs a great variety of combinations of only few instruments, aiming to explore timbre rather than to increase acoustic power. The extent to which the higher dynamics shown by Pachelbel’s canon (cf. PAC for RMS.energy in Figure 3) relate to the observed reduction of listeners self-perceived anxiety is, however, not yet clear and should be further explored.

Finally, concerning the pitch-related features, Pachelbel’s canon, characterised by the use of *basso continuo*, presents a low range of F0, whereas Schönberg’s sample, presenting orchestral instruments from a great range of registers (playing often *a solo*), shows a very large range of F0 (cf. PAC and SCH for F0 in Figure 3). This appears to be in line with the general belief that low pitches should be considered when inducing relaxation [11].

## 4. Discussion

The first objective of this work was to perform a listening experiment aimed to investigate the relaxing properties of music from different Western historical traditions. The outcomes from the user study suggest that the suitability of Classical Western music as a relaxing method, typically investigated in previous works [5,11,18,19,20], might be restricted to tonal (or even to the Baroque) music. This confirms the outcomes of [53], which showed that classical Baroque music effectively reduces anxiety induced in healthy individuals (regardless their ethnicity) as much as preferred music, i.e., music chosen by the users for having, in their opinion, relaxing properties.

However, since the authors of [53] did not control either for listeners’ familiarity or for liking, it is not clear whether these confounding factors had a role in the listeners’ decline of their self-perceived anxiety. Still, tonal music, such as Baroque music, is very present in Western culture. Due to this, we assumed that our listener group was more familiar with Pachelbel’s canon than with the other musical samples.

Considering that the results for the two participants with musical background knowledge in classical music who listened to the Schönberg’s sample were comparable to those of the listeners without such a background, we did not observe evidences of any effect on listeners’ reactions concerning familiarity or exposure in our study. Note that we assume that only the two participants with musical background might be familiar with the sample by Schönberg.

Since the modal samples, i.e., the ones by Debussy and the Gregorian chant, were shown to have less successful relaxing properties than Pachelbel’s canon but more than the music by Schönberg, we can indeed connect the musical language and familiarity with the relaxing properties of a piece. Baroque music being tonal and the most familiar to the WEIRD population is most suitable; *Expressionist* music being almost atonal and the least familiar to the WEIRD population is least suitable. Nevertheless, to really understand whether familiarity plays a role in the relaxing effect displayed by listening to Pachelbel’s canon, a Baroque sample unfamiliar to the listeners should also be assessed in future investigations.

While we consider it to be likely that all our participants were familiar with Baroque classical music, we consider it being unlikely that all of them would have chosen it as their preferred music. Taking into account preference as confounding factor, in addition to familiarity, obviously adds another level of complexity, which might not be easily handled. The most obvious solution to deal with this would be to consider non-Western users, which, however, due to the globalised world in which we live, might not guarantee non-familiarity or non-preference.

Although  the WEIRD population is not representative for the whole one, we strongly believe that this kind of investigation is still relevant, since these findings might be at least helpful for Western users and those exposed to the Western culture. Still, we acknowledge this limitation of our study and highlight the need of future interdisciplinary collaborations in order to solve this sampling bias [85]. Concerning preference, we consider unlikely that Pachelbel canon would be the preferred music in the listeners within the Baroque sample group; thus, we interpret our results in line with those by [53], who showed that Baroque music decreased self-perceived induced anxiety disregarding listeners’ preferences.

Confirming previous findings [30,55], Pachelbel’s canon was found to be associated with a decline in self-perceived anxiety in our exploratory study. Differently, the presented outcomes do not confirm the previous results for the Debussy’s sample, which was shown to be efficient in reducing anxiety in previous works [63]. One reason might be the differences in terms of anxiety and listeners between both studies, which are hardly comparable: [63] investigated the treatment of medical anxiety in hospitalised patients; we investigated to which extent listening to this musical sample impacts induced anxiety in healthy individuals.

In addition, another methodological difference between our study and the one by Bolwerk [63] is that we evaluate the effect of Debussy’s sample alone, while in [63] Debussy’s sample is taken as a part of a musical session, i.e., it was not played alone but together with a Baroque and a Classical sample (the first by Bach and the second by Beethoven).

Since the first sample of the listening session considered in [63] was the Baroque one, we could hypothesise that the relaxing effect aroused from the first piece and simply remained in the listeners while listening to the other two. Considering this, we might interpret that Debussy’s sample does not increase anxiety (the negative effect of some musical genres to increase self-perceived anxiety has been shown for example for electroacosutic music [55]); however, we cannot be completely sure about its potential relaxing effects.

The second objective of this work was to assess whether music with relaxing properties acoustically differs with respect to the one without such capability. To this end, the suitability of existing features sets tailored to emotion modelling from audio was assessed in the context of anxiety. Our evaluation of the acoustic features indicates that large feature sets, such as ComParE [49] or eGeMAPS [50], typically characterised by many redundant feature, might be unsuitable for modelling anxiety, as shown by the high overlap across musical samples varying on their potential relaxing properties. This is in line with findings aimed to identify the acoustic properties relevant to retrieve emotional dimensions from musical samples, which indicate that selected features yield better results than brute-force sets, such as ComParE [44].

The presented results also confirm the suitability of acoustic features previously identified for dimensional emotional modelling [38], for its consideration in the context of music-based treatments aimed to reduce self-perceived anxiety. Harmonicity, strongly related to the tonal components of a musical piece, appears to be related to the relaxing properties of the evaluated samples; this confirms our interpretation of tonal music as potentially suitable to induce relaxation.

In addition to harmonicity, the relationship of the spectral flux and the relaxing properties of the evaluated samples might be explained by the importance of timbre to reducing anxiety. Indeed, previous works have indicated that specific timbres, such as strings, might be more appropriate to inducing relaxation than others [11]—information that might be captured by the spectral flux.

## 5. Conclusions and Future Work

By assessing the relaxing properties of a variety of Western musical traditions, our work shows that only listeners exposed to the Baroque sample displayed a decrease in their self-perceived anxiety. This indicates that the capability of Classical Western music to reduce listeners’ distress, as already acknowledged in previous works [5,11], should probably be restricted to tonal music.

The presented research reveals that the evaluated acoustic parameters, which have been identified as suitable to identify emotional content from music [38], appear to be appropriate in retrieving important information concerning music’s relaxation properties. Nevertheless, more research is required to understand the role of some of them, for instance, concerning the role of dynamics.

In particular, our outcomes also suggest that the presence of a tonal centre, likely related to a listeners’ sensation of familiarity, is a musical criterion that should be taken into account when using music to reduce anxiety. This musical property can be mapped onto acoustic features, such as harmonicity, which was found to be relevant in our evaluation.

Following the findings presented, the next step should be to evaluate a variety of tonal samples with different scoring. By considering only tonal music, this will enable evaluation of whether the vocal and orchestral samples played a role in our study due to the scoring itself or due to the lack of a tonal centre.

In addition, the role of listeners’ musical preferences in anxiety reduction should also be evaluated in future work. Taking into account the broad nature of users’ musical tastes, evaluating listeners’ musical preference will extend the research questions presented to a more complex dimension where musical genre should be carefully considered to group participants; this will prevent this variable from being a confounding factor.

Assessing a variety of musical styles has been identified as a particularly relevant research direction when investigating induced stress [11]. This will certainly create further connections to emotion-related aspects, such as a listener’s personality [86].

Finally, as the *emotivist position* has been rarely considered when assessing musical emotions with methods from signal processing, further research investigating the acoustic parameters involved in evoking listeners’ felt emotions should be carried out. This will pave the way for the application of signal processing and computational methods to healthcare, a knowledge domain that will not only encourage developments in psychology but will also promote multidisciplinary research, such as the development of music recommender technology for therapeutic applications.

Since MIR in general and MER in particular present a strong bias towards a terminology and an understanding rooted in Western culture, future research should also put effort in assessing repertoires from other cultures, for which the modes of listening and underlying assumptions used to interpret musical emotions will surely differ [87]. As choosing the emotional taxonomy used to annotate the musical excerpts is the first step in designing MER models [88], research beyond the Western culture will likely call for an adaptation of existing methods and measurement instruments [89], e.g., in order to handle emotional terms that are difficult (or even impossible) to translate.

## Figures and Tables

**Table 1:** Description and number of Low Level Descriptors (LLDs) for each of the acoustic feature sets: EmoMusic, ComParE, eGeMAPS, and  NoAnx.

	Description	LLDs
EmoMusic	Eight descriptors: roll off, sharpness, spectral centroid,	8
	energy, harmonicity, loudness, F0, spectral flux	
ComParE	Four types of descriptors: spectral (41), Mel-Frequency Cepstral	65
	Coefficients—MFCCs (14), prosodic (5), sound quality (5)	
eGeMAPS	Three types of descriptors: spectral (7), frequency (11),	25
	energy/amplitude (7)	
NoAnx	Eleven descriptors: roll off, sharpness, spectral centroid,	11
	energy, harmonicity, loudness, F0, spectral flux,	
	alpha ratio, Hammaberg index, MFCC2	

**Table 2:** Tukey post-hoc results for the multiple comparisons from the ANOVA between the control group and the listening conditions: Pachelbel, Gregorian, Debussy, and Schönberg. The mean (μ) and standard deviation (σ) of the difference between pre- and post-condition per sample, mean differences (Diff) with respect to the control group, lower and upper confidence intervals (lwr and upr), *p*-value, and Cohen’s *d* are given.

	μ	σ	Diff	lwr	upr	*p*	*d*
Control	0.2	1.62	−	−	−	−	−
Pachelbel	3.2	2.53	3.0	0.45	5.55	0.013	0.55
Gregorian	1.5	2.27	1.3	−1.25	3.85	0.601	0.12
Debussy	1.2	1.81	1.0	−1.55	3.55	0.798	0.06
Schönberg	0.6	1.65	0.4	−2.15	2.95	0.991	0.00

**Table 3:** Results for the Welch-ANOVA and Games–Howell (post-hoc test) on the NoAnx feature set considering Pachelbel’s canon as reference for the pairwise comparisons with the other samples: Gregorian chant, Debussy’s sample, and  Schönberg’s sample. For the ANOVA, F score, degrees of freedom (df1 and df2), and epsilon squared ($\epsilon^{2}$) are indicated. For Games–Howell, the mean difference (Diff) and Hedge *g* are also given. Results are indicated for the three feature groups: timbre, dynamics, and pitch.

Feature	Welch				Games–Howell Post-Hoc					
	ANOVA				Gregorian		Debussy		Schönberg	
	F	df1	df2	$\epsilon^{2}$	Diff	*g*	Diff	*g*	Diff	*g*
*Timbre*										
Roll off	89.0	3	1129	0.21	36.4	0.32	10.8	0.07	224.3	1.03
Sharpness	93.7	3	1129	0.20	0.02	0.36	−0.01	0.10	0.12	1.02
Centroid	81.1	3	1129	0.18	9.00	0.11	−19.3	0.17	130.2	0.89
Harmonicity	261.8	3	1129	0.60	−0.15	0.65	−0.42	3.10	−0.43	3.34
MFCC	126.4	3	1129	0.25	5.20	0.78	1.91	0.24	−7.39	0.90
*Dynamics*										
RMS.energy	679.1	3	1129	0.60	−0.01	1.20	−0.04	4.37	−0.05	5.48
Loudness	529.9	3	1129	0.57	−0.30	0.83	−0.94	3.81	−0.97	4.30
*Pitch*										
F0	437.9	3	1129	0.28	96.8	3.50	15.2	0.30	85.1	0.88
Spec.Flux	462.5	3	1129	0.60	−0.08	0.66	−0.27	3.20	−0.31	4.18
Alpha.Ratio	54.0	3	1129	0.11	−3.61	0.87	−2.86	0.57	0.74	0.14
Hammarberg	59.9	3	1129	0.10	6.01	0.99	7.26	1.05	4.85	0.78

## Data Availability

For reproducibility, we make the R code, acoustic features, and the anonymous data freely accessible in this public repository: https://github.com/SEILSdataset/Music_Anxiety_Acoustic (accessed on 5 December 2021).
